# Internal health locus of control in users of complementary and alternative medicine: a cross-sectional survey

**DOI:** 10.1186/1472-6882-14-320

**Published:** 2014-08-30

**Authors:** Lena Schützler, Claudia M Witt

**Affiliations:** Institute for Social Medicine, Epidemiology and Health Economics, Charité - Universitätsmedizin Berlin, Berlin, Germany; Institute for Complementary and Integrative Medicine, UniversityHospital Zurich, Zurich, Switzerland

**Keywords:** Internal-external control, Complementary therapies, Cross-sectional studies

## Abstract

**Background:**

Complementary and alternative medicine (CAM) is widely used in Germany, with some treatments eligible for health insurance reimbursements. CAM encourages patients to play an active role in their healing process. The belief that a person’s own behavior influences health is assessed as the internal health locus of control (IHLOC). Studies on the association between IHLOC and CAM use yield inconsistent results. Using various indicators of CAM use, we evaluated whether there were differences in IHLOC between different groups of CAM users.

**Methods:**

A cross-sectional online survey was conducted. IHLOC was compared between participants with high and low appraisal of CAM, between participants who used different types of medications (none, CAM, conventional, both), and who consulted with different health care professionals (none, CAM, conventional, both). Independent samples t-tests and ANOVAs were conducted for the total group and for subgroups of chronically ill and healthy participants. Post-hoc, we conducted a multivariate linear regression evaluating which indicators of CAM use or other characteristics showed the strongest association with IHLOC.

**Results:**

A total of 1,054 undergraduate students completed the survey. Participants with high CAM appraisal showed higher IHLOC than those with low CAM appraisal, regardless of whether they were chronically ill (p < .001). Participants without chronic conditions showed higher IHLOC when only using CAM medications than when using either conventional medications alone or both conventional and CAM medications (p < .05). All participants showed higher IHLOC when visiting only CAM practitioners than when visiting either only conventional or both conventional and CAM practitioners (p < .05). CAM appraisal was associated the strongest with IHLOC in the linear regression model.

**Conclusions:**

Generally, participants using CAM more or exclusively, and participants with higher appraisal of CAM showed higher IHLOC than those with less CAM use or lower CAM appraisal. Because of the cross-sectional design, it is not possible to determine whether differences in IHLOC are reasons for or consequences of CAM use. Research using a longitudinal design is needed. The sample, though more representative than most student samples, might not represent the general population. Studies evaluating clinical populations might add to the findings.

## Background

Therapies belonging to the spectrum of complementary and alternative medicine (CAM) are widely used in Germany [1, 2] and worldwide [3, 4]. According to a recent systematic review, [5] herbal medicine and chiropractic care are the most popular CAM treatments in Germany, however, the data differs depending on the definition of CAM and the CAM professionals included (physicians only or also lay practitioners). While in Germany, some CAM treatments, such as acupuncture for chronic low back pain and osteoarthritis of the knee, are generally reimbursed, it is different in other countries where reimbursement depends on the patient’s insurance. The association between the use of CAM and its users’ characteristics and beliefs has been investigated in numerous studies (e.g., [6–8]). One of the characteristics that has been examined repeatedly is internal health locus of control (IHLOC). IHLOC describes the perception that one can influence one’s health (as opposed to health being determined by powerful others, e.g., physicians, or by chance, fate, or God [9, 10]). Barrett et al. interviewed CAM practitioners about their views on health and health care, coming to the conclusion that the patients’ responsibility for their own health is an important part of CAM [11]. The patient works together with the health care professional to improve his or her health. Often this involves changing aspects of one’s lifestyle (e.g., diet or sleeping behavior), or practicing certain techniques (e.g., breathing or meditating). Taking responsibility for their recovery in this way might be one of the reasons patients choose CAM in the first place.

In a systematic review of beliefs of CAM patients, the majority of studies found correlations between the use of CAM and the desire for personal responsibility and health empowerment in general [7]. Qualitative studies come to that conclusion as well [12, 13]. However, when looking at IHLOC in the same review, only three studies [14–16] showed positive correlations between internal locus of control and CAM use, and ten showed no relationship [7]. However, this result must be regarded with caution as most of the studies included in the review used a measure for IHLOC [17] that has questionable reliability [18]. Additionally, the assessment of CAM use varies from study to study, including giving the participants a list of CAM modalities and defining everybody as a CAM user who has used at least one of these modalities, and recruiting participants at CAM-based treatment centers and comparing them to participants recruited at GP-based treatment centers. More recent studies, not included in the above review, also come to ambiguous results regarding associations between CAM use and IHLOC. No relationship between internal locus of control and requests for CAM was found in an inpatient sample in Germany [19]. However, positive relationships have been found in cancer patients [20, 21], in patients with lower back pain [22], in the healthy population [23], and in a mixed sample [24]. The picture, therefore, remains unclear and warrants further investigation.

The aim of this study was to evaluate differences in IHLOC between participants with high and low appraisal of CAM, with different patterns of medication use (none, only CAM, only conventional, or both), and with different patterns of health-care consultations (none, only CAM practitioners, only conventional practitioners, or both) using a large sample.

## Methods

### Subjects and Setting

The study was designed as an online survey conducted at a university (FernUniversität in Hagen) that was also used for the validation of a scale to measure body-efficacy expectation (BEE) described in a different publication [25]. In this manuscript, we report the methods relevant to this publication; for overall methods, see Schützler and Witt, 2013 [25].

As an anonymous survey, the study did not require the approval of an ethics committee.

### Measurement Instruments

#### Internal Health Locus of Control (IHLOC)

IHLOC [9, 10, 26] was assessed with the seven items of the “internal” subscale of the German version of the multidimensional health locus of control questionnaire [27]. Items assess the extent to which people believe that they are responsible for their health, e.g., “If I take the right actions, I can stay healthy”. The items are rated on a 6-point Likert scale ranging from 1 (strongly agree) to 6 (strongly disagree). To calculate the total score, the scores are reversed so that higher total scores represent higher IHLOC values. Because the manual for the questionnaire does not contain instructions for dealing with missing values, an IHLOC score was only computed for participants who had no missing values. Cronbach’s alpha of the scale in our sample was .84.

### Chronic conditions

Participants were asked if they had a chronic condition and, if so, what chronic condition they had. They were also given a list with acute conditions and were asked to check any condition they suffered from in the last six months as well as the duration of the condition. If the duration was >100 days, the condition was considered to be chronic and was reclassified as such [25].

### Appraisal of CAM

Participants were presented with a list of CAM treatments (including the option to specify three more CAM treatments not on the list) and were asked to answer one of the following: “I have positive experience with this treatment”, “I have negative experience with this treatment,” “I can imagine this treatment is effective, but have not tried it yet”, “I do not think this treatment is effective”, or “I do not know about this treatment/no opinion”. The number of treatments that participants reported having had positive experiences with or regarded as effective even though they had not tried them personally was used as a surrogate measure for positive CAM appraisal (appraisal score, see also [25]).

### Medication profile

Participants were given a list of prescription and over-the-counter drugs and reported if they had used them in the six months prior to the study. These included: allopathic drugs (e.g. but not limited to pain killers, antibiotics, detumescing nasal sprays, beta-blockers, drugs for thyroid dysfunction, ointments) and psychiatric drugs, together regarded as conventional medications, phytomedicine, homeopathic drugs, TCM herbs, Bach flowers, and essential oils, regarded as CAM medications. Participants could also list other medications that were then screened and classified as either conventional or CAM medications. We assigned the participants to one of the following groups: (1) uses only CAM medications, (2) uses only conventional medications, (3) uses both CAM and conventional medications, and (4) uses no medications at all [25].

### Consultation profile

Participants were given a list of physicians and health practitioners and reported if they had visited them in the six months before the study: general practitioners or specialists (e.g. but not limited to internists, orthopedic specialists, gynecologists, otorhinolaryngologists, eye specialists, etc.), together regarded as conventional consultants, naturopathic MDs, homeopathic MDs, Traditional Chinese Medicine (TCM) MDs, anthroposophic medicine MDs and non-medical practitioners (German “Heilpraktiker”), together regarded as CAM consultants. They could also list other practitioners that were screened and classified as either conventional or CAM consultants. We then assigned the participants to one of the following groups: (1) only sees CAM consultants, (2) only sees conventional consultants, (3) sees both CAM and conventional consultants, and (4) does not consult with any health care professionals [25].

### Statistical methods and analyses

The sample was split into two subgroups based on the CAM appraisal score resulting in one subgroup with low appraisal and one with high appraisal of CAM. An independent samples t-test was conducted to compare IHLOC in both subgroups.

One-factor ANOVAs were used to compare IHLOC of the participants according to their medication and consultation profiles (for more details see [25]). Orthogonal linear contrasts were used to compare pre-defined groups: we compared IHLOC of participants without any medication use with all other participants, and participants who only used CAM medications with those who only used conventional, or both CAM and conventional, medications. Accordingly, we compared IHLOC of participants reporting no consultations with any health care professionals with all others, and participants who only visited CAM practitioners with those who only visited conventional, or both CAM and conventional, practitioners.

To check if participants with and without chronic conditions differed in their IHLOC, an independent samples t-test was conducted to compare the two groups. If significant, all analyses were conducted for the subgroups with and without chronic conditions in addition to the analysis of the total sample.

In addition to these analyses, a multivariate linear regression model was built to evaluate which variables contributed the most to high or low IHLOC when considering them all together. The IHLOC score was the outcome and the following variables were added to the model as predictors: sex, age, presence of a chronic condition, appraisal score (as a numerical variable), and medication and consultation profiles. Factors of the latter were dummy-coded, and taking only conventional medication or visiting only conventional practitioners served as the reference category. No selection of predictors was conducted, rather, all predictors were left in the model, regardless of whether they were significant or not. The variance inflation factor was computed for all variables to check for multicollinearity.

All computations were performed using the IBM Statistical Package for the Social Sciences (SPSS), Version 20 for Windows. Because statistically significant differences in a large sample are not necessarily relevant, we computed effect sizes for the t-tests and ANOVAs using the program G*Power 3.1 [28].

## Results

**Figure 1:**
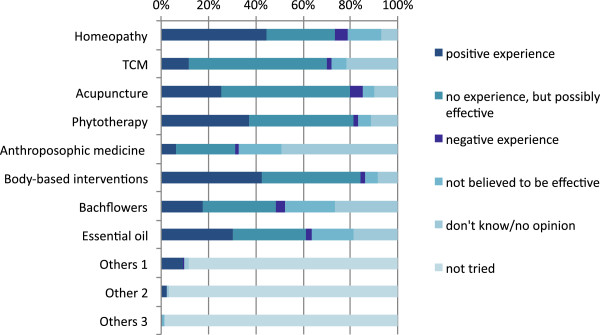
**Experience with complementary and alternative medicine.**

A total of 1,054 students (mean age 32.74 years, SD = 9.32) completed the survey; 80.4% of the sample were women, 17.9% were men, and 1.7% did not indicate their gender; 34.8% of the participants reported a chronic health condition; and 77.3% of the participants had tried at least one CAM treatment. With the majority of CAM treatments, the participants had positive experiences, or at least assumed them to be effective (Figure 1). IHLOC scores were missing for 3.2% of the sample. Because this number is relatively small we decided to not impute missing values.

We used Q-Q plots to check the normality assumptions for the t-tests and ANOVAs. Only slight deviations from normality were found so we decided to conduct parametric tests. T-tests and ANOVAs are relatively robust against violations of normality assumptions as long as the sample size is large enough. Additionally, most studies evaluating IHLOC use comparisons of means which makes comparisons between studies difficult when conducting different analyses.

Participants without chronic conditions had higher IHLOC (M = 29.96, SD = 4.53) than participants suffering from a chronic condition (M = 28.32, SD = 5.46; p < .001, effect size d = .33). Therefore, all of the following analyses were computed for the total sample, and separate analyses were performed for participants with and without chronic conditions. Appraisal scores were between 0 and 11 with a mean of 5.32 (SD = 2.40) and a median of 6. The sample was split into one group with low CAM appraisal (scores of 0–5) and one with high CAM appraisal (scores of 6–11). This corresponds to a split where one group has scores below the mean and the other has scores above the mean. A median split, which would have been the normal procedure for planned group comparisons, was not feasible because 159 participants had a score of 6, i.e., the median itself. The low appraisal group had a mean appraisal score of 3.20 (SD = 1.64) and the high appraisal group had a mean appraisal score of 7.21 (SD = 0.96). IHLOC differed significantly in these groups, with higher values observed in participants with a high appraisal of CAM. This was true both for the total sample (M_High Appraisal_ = 30.10 (SD = 4.80), M_Low Appraisal_ = 28.47 (SD = 4.92), p < .001, d = .34) and for the subgroups with a chronic condition (M_High Appraisal_ = 29.27 (SD = 5.08), M_Low Appraisal_ = 27.17 (SD = 5.70), p < .001, d = .39) and without a chronic condition (M_High Appraisal_ = 30.59 (SD = 4.60), M_Low Appraisal_ = 29.24 (SD = 4.34), p < .001, d = .30). According to Cohen’s conventions for standard mean differences, effect sizes of .2, .5 and .8 correspond to small, medium and large effects [29]. Therefore, the effects were small to moderate as is reflected in how they correspond to small numerical differences on the IHLOC scale.

Participants who only used CAM medications showed the highest IHLOC results, followed by participants who did not use any medications, those who used both CAM and conventional medications, and lastly those who only used conventional medications. This pattern was found in the total sample as well as in the subgroups of participants with and without chronic conditions. ANOVAs comparing the participants according to their medication use showed significant differences with a small effect size for the total sample, but not for the subgroups of participants with and without chronic conditions (Table 1). The linear contrasts showed that the overall effect was due to significantly higher IHLOC scores in participants who only used CAM medications compared to those who only used conventional or both CAM and conventional medications, while the difference between those who did not use any medications at all and all other participants was not significant (Table 2). This was also true for participants without chronic conditions, but not for those with a chronic condition (Table 2).

**Table 1:** **IHLOC according to medication use in the past six months**

	Total group		Chronic condition		No chronic condition	
	N	Mean (SD)	N	Mean (SD)	N	Mean (SD)
CAM medication only	98	30.74 (4.92)	23	29.87 (5.43)	75	31.01 (4.76)
Conventional medication only	341	28.87 (5.03)	143	28.01 (5.55)	197	29.49 (4.54)
Both	313	28.95 (4.64)	151	28.11 (4.99)	160	29.80 (4.13)
No medication	268	29.89 (4.99)	40	29.30 (6.70)	219	30.13 (4.67)
Total	1020	29.34 (4.93)	357*	28.32 (5.46)	651*	29.96 (4.53)
F (df)		5.52 (3,1016)		1.27 (3,353)		2.25 (3,647)
p		<.01		.284		.081
Effect size f		.13		.11		.10

*Sizes of subgroups do not add up to 1020 because of missing data regarding the presence/absence of a chronic condition.

**Table 2:** **Subgroup comparisons regarding medication**

	Total group			Chronic condition			No chronic condition		
	Difference in IHLOC	t (df)	p	Difference in IHLOC	t (df)	p	Difference in IHLOC	t (df)	p
No medication vs. any medication	1.10	1.00 (1016)	.32	1.90	.66 (353)	.51	.08	.07 (647)	.94
CAM medication only vs. CAM and conventional or conventional medications only	3.67	3.46 (1016)	<.01	3.62	1.53 (353)	.13	2.74	2.39 (647)	<.05

Participants who only consulted CAM practitioners showed the highest IHLOC results, followed by those who did not see any practitioners, those who consulted both CAM and conventional practitioners, and lastly, those who consulted only conventional practitioners. This pattern was found in the total sample as well as in the subgroups of participants with and without chronic conditions (Table 3). ANOVAs comparing the participants according to the health care professionals they visited yielded significant overall results in the total sample as well as in the subgroups of participants with and without chronic conditions (Table 3). The effects were of moderate size. The subgroup analyses showed that this overall result was due to a significantly higher IHLOC in participants who only visited CAM practitioners compared to those who visited both conventional and CAM practitioners or conventional practitioners only. This was found in the total sample and both subgroups (Table 4).

**Table 3:** **IHLOC according to health-professionals’ consultation during the past six months**

	Total group		Chronic condition		No chronic condition	
	N	Mean (SD)	N	Mean (SD)	N	Mean (SD)
CAM consultation only	24	32.58 (4.24)	8	34.00 (4.28)	16	31.88 (4.18)
Conventional consultation only	517	28.44 (4.96)	229	27.47 (5.36)	286	29.23 (4.49)
Both	78	29.08 (4.80)	42	28.83 (5.06)	35	29.49 (4.53)
No consultation	401	30.37 (4.67)	78	29.96 (5.39)	314	30.57 (4.48)
Total	1020	29.34 (4.93)	357*	28.32 (5.46)	651*	29.96 (4.53)
F (df)		15.78 (3,1016)		7.62 (3,353)		5.62 (3,647)
p		<.001		<.001		<.01
Effect size f		.22		.25		.16

*Sizes of subgroups do not add up to 1020 because of missing data regarding the presence/absence of a chronic condition.

**Table 4:** **Subgroup comparisons regarding health professionals consultations**

	Total group			Chronic condition			No chronic condition		
	Difference in IHLOC	t (df)	p	Difference in IHLOC	t (df)	p	Difference in IHLOC	t (df)	p
No consultation vs. any consultation	1.00	.74 (1016)	.46	-.42	-.15 (353)	.88	1.13	.72 (647)	.47
CAM consultation only vs. CAM and conventional or conventional consulations only	7.65	3.73 (1016)	<.001	11.70	3.03 (353)	<.01	5.04	2.12 (647)	<.05

The variance inflation factors for all variables were approximately 1, and the highest value was 1.6, indicating little multicollinearity. The linear regression model showed that the appraisal score (beta = .15, p < .001), the presence of a chronic condition (beta = -.11, p < .001), not visiting any health-care professionals (beta = .17, p < .001), and visiting only CAM practitioners (beta = .11, p < .01) were significant predictors for IHLOC. Suffering from a chronic condition was associated with a lower IHLOC, while the three others were associated with a higher IHLOC. All other predictors were not significant (Table 5). Despite the highly significant predictors, the explained variance in IHLOC was only 9%.

**Table 5:** **Results of the linear regression model predicting internal health locus of control (IHLOC)**

Predictor	B (95% CI)	Beta	P
Appraisal score	.32 (.18, .45)	.15	<.001
Chronic condition	-1.18 (-1.84, -.52)	-.11	<.001
Sex: female	-.18 (-.96, .60)	-.01	.65
Medication [Reference: only conventional medication]			
Only CAM	.36 (-.77, 1.49)	.02	.53
Both CAM and conventional	-.01 (-.78, .75)	-.00	.97
None	-.07 (-.91, .77)	-.01	.86
Consultations [Reference: only conventional practitioners]			
Only CAM	3.49 (1.50, 5.48)	.11	<.01
Both CAM and conventional	.40 (-.78, 1.59)	.02	.50
None	1.73 (1.02, 2.43)	.17	<.001
Age	.01 (-.02, .05)	.02	.44

## Discussion

We aimed to determine whether IHLOC differed according to CAM appraisal and the use of different medications (only CAM, both conventional and CAM, only conventional, or none) or chosen health care professionals (only CAM, both conventional and CAM, only conventional, or none). Because the study was observational, one must keep in mind that the study population is often heterogeneous, with the CAM group including more patients with chronic illnesses. It has been shown that poor health status is a predictor for CAM use [6]. Additionally, a chronic condition might lead to lower IHLOC results because it has been shown that IHLOC scores are higher in healthier subjects [30]. Indeed, in our sample the participants suffering from chronic conditions showed lower IHLOC than participants without chronic conditions. Therefore, we controlled for possible confounding by conducting subgroup analyses of participants with and without chronic conditions.

The proportion of participants who had used at least one CAM treatment was higher than previously reported for the general German population [2, 31]. However, our study population was a student population whose social statuses might be higher than that of the general population. A higher social status has been found to be a predictor of CAM use [1, 31]. The proportion of participants with a chronic condition was comparable to that of the general German population [32].

Higher appraisal of CAM was moderately related to higher IHLOC, regardless of whether participants had a chronic condition. Focusing on the medication use of the participants, differences were found between those participants who only used CAM medications and all others; however, not for the chronically ill participants. This indicates that the presence of a chronic condition is a more important predictor of IHLOC than the type of medication used. For the type of health care professional the participants consulted, there were differences between the participants who only consulted CAM practitioners and all others. This was even true for the group with a chronic condition. The results seem to support those studies that found IHLOC to be positively related to CAM use [14–16, 20–24].

When considering CAM appraisal, medication and consultation profiles, chronic conditions as well as sex and age in one model, we found that CAM appraisal had the strongest association with IHLOC, followed by suffering from a chronic condition (negative association), not visiting any health care professionals or only visiting CAM practitioners. However, all of the variables combined only explained 9% of the variance in IHLOC. This indicates that there are other factors associated with IHLOC that were not included in our model. We only assessed whether participants had a chronic condition; however, how they manage it might be more important regarding IHLOC. This points to self-efficacy expectations [33] as one important factor.

Strengths of our study include the large, heterogeneous sample. As described in the validation publication of the BEE scale [25], this was not an ordinary student sample: the FernUniversität in Hagen is the only distance learning university in Germany. Students vary considerably in age, lifestyles, and previous knowledge and experiences. The university has no grade point enrollment cut-off, and many students have work experience or work during their course of studies (in fact, 80% do so, [34]), and many have children.

CAM use was assessed in several ways: We used an appraisal score that included positive experiences with CAM as well as positive assumptions regarding CAM modalities that have not been tried by the participants themselves. Furthermore, we looked at the types of medications the participants used and the health care professionals they visited for their health problems. Additionally, we took care to account for the role of chronic conditions as a possible confounder by running subgroup analyses for all comparisons. IHLOC was assessed using a widely used and well-validated instrument [9, 10].

For non-significant mean differences, the trends also pointed in the assumed directions. The lack of significant differences in the subgroup of participants with a chronic condition is likely due to the smaller sample size (approximately 35% of the total sample).

Several limitations of this study must be discussed. First, because of the cross-sectional study design, there is no way to determine whether a higher IHLOC is a disposition that makes people prone to using CAM, or whether it is a result of positive experiences with CAM treatments. In a study by Hoffmann et al. [35] concerning changes that occur during CAM treatment, an increase in IHLOC was observed during inpatient integrative medicine treatment of patients with chronic conditions. However, the study lacked a control group. In the interview study by Cartwright and Torr [36], the authors describe how the participants adopt concepts from CAM when they refer to “balance”, “qi”, “energy”, etc. (p. 564), i.e., ideas and theories have been communicated between them and their practitioner. Such results point out the possible change of beliefs and expectations during the course of treatment. However, it is generally assumed that people chose CAM because it is in accordance with beliefs that they already hold [7, 37]. It would be worthwhile to investigate IHLOC and CAM in a longitudinal design, i.e., assessing participants’ IHLOC before and after a CAM treatment. In that way, one could detect changes in IHLOC as well as possible differences between different CAM treatments (for example, yoga‘s very active patient role, and homeopathy’s more passive patient role).

Second, health statuses and chronic conditions were only assessed through self-reports. Analyzing samples with a confirmed diagnosis might be worthwhile to come to a final conclusion regarding whether health status is a confounder or not. Additionally, the CAM-related variables might not be optimal, as discussed elsewhere [25].

Third, although the sample was more heterogeneous than student samples generally are, whether the results can be generalized to the general population remains up for debate. Also, results may be different in countries with different reimbursement strategies of CAM treatments since the status of CAM might be different in such countries.

Methodologically, using the same sample that was used in the BEE scale validation study makes this study somewhat exploratory. Additionally, in the ANOVAs and contrast analyses, the sample sizes of the compared groups were quite different for some groups, especially for those of participants using only CAM medications or practitioners, which included very few participants. This is a serious problem and should be addressed in further studies by recruiting part of the sample in different settings, e.g., outpatient CAM departments or practices. In addition to that, an alpha of 0.05 was assumed in all statistical tests, resulting in multiple testing. The results should therefore be interpreted with caution regarding statistical significance, the more as only small to moderate effects could be found. Additionally, the proportion of explained variance in IHLOC when using CAM appraisal, medication and consultation profiles as well as other possibly confounding variables, such as chronic conditions, was very low. This is in accordance with the rather small effects found in the t-tests and ANOVAs.

In discussing the results, an important question has recently been posed by Lindeman [38]: are there predictors of CAM use that underlie the factors and beliefs commonly assumed to be related to CAM use? Her study used a regression model with factors that have been investigated with regard to CAM use in numerous studies, e.g., desire for control, health, education, gender, certain world views, etc. In addition to that, intuitive thinking, core knowledge confusions, and paranormal beliefs were added as predictors. Those three predictors explained 34% of the variance in CAM beliefs while all other predictors added no more than 4% of the explained variance.

Therefore, the question is justified as to whether focusing on constructs such as locus of control is worth further pursuit or if more general, underlying constructs should be increasingly taken into account.

While that question remains debatable, our results indicate that there are, in fact, differences in IHLOC when comparing different groups of CAM users.

## Conclusion

We found that IHLOC was related to CAM use by looking at several indicators for CAM use. It remains unclear whether people use CAM because it is in accordance with their higher IHLOC or if their IHLOC changes during CAM treatments. This question will have to be evaluated using longitudinal studies or structural equation modeling. Additionally, the role of possible confounders should be further illuminated.

## Abbreviations

CAM: Complementary and alternative medicine
BEE: Body-efficacy expectation
IHLOC: Internal health locus of control
TCM: Traditional Chinese Medicine.
